# The Effect of Neural Noise on Spike Time Precision in a Detailed CA3 Neuron Model

**DOI:** 10.1155/2012/595398

**Published:** 2012-06-24

**Authors:** Eduard Kuriscak, Petr Marsalek, Julius Stroffek, Zdenek Wünsch

**Affiliations:** ^1^Department of Physiology, First Medical Faculty, Charles University in Prague, Albertov 5, 12800 Praha 2, Czech Republic; ^2^Department of Pathological Physiology, Medical Faculty, Charles University in Prague, U nemocnice 5, 12853 Praha 2, Czech Republic; ^3^Faculty of Biomedical Engineering, Czech Technical University in Prague, Nam. Sitna 3105, 27201 Kladno, Czech Republic

## Abstract

Experimental and computational studies emphasize the role of the millisecond precision of neuronal spike times as an important coding mechanism for transmitting and representing information in the central nervous system. We investigate the spike time precision of a multicompartmental pyramidal neuron model of the CA3 region of the hippocampus under the influence of various sources of neuronal noise. We describe differences in the contribution to noise originating from voltage-gated ion channels, synaptic vesicle release, and vesicle quantal size. We analyze the effect of interspike intervals and the voltage course preceding the firing of spikes on the spike-timing jitter. The main finding of this study is the ranking of different noise sources according to their contribution to spike time precision. The most influential is synaptic vesicle release noise, causing the spike jitter to vary from 1 ms to 7 ms of a mean value 2.5 ms. Of second importance was the noise incurred by vesicle quantal size variation causing the spike time jitter to vary from 0.03 ms to 0.6 ms. Least influential was the voltage-gated channel noise generating spike jitter from 0.02 ms to 0.15 ms.

## 1. Introduction

The neuronal output represented by spike trains fired by individual neurons is a result of coding processes over the neuronal input and noise interfering with neuronal computation. Three ways of neuronal representation or neural coding are widely accepted by means of which spike trains, made up of individual action potentials (APs), transmit information over axons and present it to the next layer of computational units, the afferent neurons. The first one assumes information in spike trains to be represented via the mean rate of APs and neglects the relevance of the precise firing of individual APs [1–4]. The other two relate the capacity of neural coding to the precision of AP timing, either on the absolute time scale on which individual APs occur or on the relative time scale represented by interspike intervals (ISIs) separating the APs [5–7]. The time scale on which information is encoded by individual APs has long been a subject of discussion [8–14]. It has been shown that the timing of APs evoked by sensory stimuli in various subcortical sensory pathways can have precision of milliseconds [15–18], down to hundreds [19, 20], and even tens of microseconds [21, 22]. Recent studies also indicate that AP timing in the visual system could be even more precise than the relevant time scales of natural vision [23].

Growing experimental and computational effort thus emphasizes the role of the millisecond precision of neurons as an important coding mechanism for transmitting and representing information in the nervous system [24]. There is some evidence to suggest that other neural systems may utilize the temporal coding that derives from the strong temporal association between stimuli and neuronal responses seen in sensory systems. Neuronal systems like the hippocampal formation can exhibit similar AP time fidelity [25]. However, the reliability, precision, and reproducibility of neuronal responses are studied in these nonsensorial structures much less frequently, mainly because of the elusive relationship between the complex cognitive tasks they perform and the spike trains they produce. Among methods analyzing the spike train precision in these structures, a computational approach seems to be a very promising one, enabling the control of processes commonly considered as neuronal noise. In particular, neurons *in vivo* are constantly bombarded by background synaptic activity, by the so-called synaptic background noise, encompassing the highly-complex, sustained and irregular firing of presynaptic neurons [13, 26–28]. In some studies, the synaptic background noise is treated as a source of neural randomness. This is frequently due to the fact that the presynaptic neurons are not under complete control of experimental conditions. This way many conclusions regarding the spike train randomness may be challenged.

Other sources of noise, also incurred by synapses, reside in the probabilistic nature of synaptic vesicle release. Many central synapses, for example, those in hippocampal area CA3 (the third area of Cornu Ammonis), possess on average only one release zone with the probability of release of one synaptic vesicle ranging from 0.1 to 0.9 [29, 30]. The variation of vesicle quantal size [31, 32] and the stochastic opening of postsynaptic ligand-gated channels make up the other important sources of noise, causing the amplitudes of postsynaptic current to vary from one event to the next.

On excitable membranes the voltage-gated channel noise, caused by random fluctuations of voltage-gated ion channel states, can be the most dominant source of noise [33–36], especially in axons, significantly increasing the spike time jitter [37, 38]. The thermal Johnson noise [33], originating in the thermal agitation of ions in an electrical conductor, is thought to have negligible effect on the spike time precision [13, 39].

Summarizing the experimental and computational effort, recent evidence has accumulated supporting the presence of temporal coding in many regions of the nervous system. In sensory systems new experimental techniques enable spike train precision to be estimated very accurately for various stimulation paradigms. Nevertheless, for the reasons mentioned above, fewer data are available to confirm temporal coding in nonsensorial systems [25, 40–42]. Therefore we took advantage of the computational approach and simulated a realistic multicompartmental model of the CA3 pyramidal neuron under different stimulation schemes and subject to various sources of neuronal noise. The aim of our effort was to assess on which time scales and under which conditions the CA3 neuron can generate high-fidelity temporal coding.

## 2. Methods

For a study with a neural model, a model of complexity corresponding to the outlined aim has to be used. Simplest cell models, working like all-or-none-response binary units, are usually employed in simulations analyzing global behavior of neural networks containing a lot of neurons. Neural models of medium complexity levels are represented by one or just a few variables governing the dynamics of the membrane potential, where the spatial extents of the neuron (and therefore all spatial coordinates and variables) are neglected. These are used to model the behavior of local neural circuits and are commonly built and simulated using the compartmental approach. Compartmental models studying the input-output characteristics of single neurons are called detailed compartmental models. They implement the detailed electrotonic structure of the membrane of any desired tree topology, including complex ion channel dynamics and other intrinsic mechanisms controlling the single-cell behavior [43]. One example of the last category is the model used in our study.

We used the multicompartmental model of a rodent CA3 pyramidal neuron created in the GENESIS software environment [44, 45]. The main properties of the model were adapted from the well-established multicompartmental CA3 model of a guinea-pig hippocampal pyramidal neuron [46] and also from other sources [47], for the full list of relevant references and respective parameters (see Supplementary (Table 1) available online at doi: 10.1155/2012/595398).

The electrotonic structure consisted of 64 somadendritic compartments and four axonal compartments. Voltage-gated and synaptic conductances were modeled and distributed according to the available data (see Supplementary Table 1) [48–56]. The model contained seven types of voltage-gated conductances, four types of ligand-gated conductances, and its input was modeled via various groups of presynaptic neurons. It reproduced several firing regimes of CA3 pyramidal neurons, including the response to somatic or dendritic current injection and orthodromic stimulation, antidromic spikes and bursts [45, 46].

### 2.1. Synaptic Conductances and Noise

7874 synapses were attached to the somadendritic membrane of the CA3 pyramidal neuron used in our simulations. Four types of synaptic conductances received their input from 11 different groups of presynaptic neurons firing at four different frequencies (see Supplementary Table 1). Each presynaptic neuron was modeled as a statistically independent, randomly firing spike generator controlled by a Poisson point process. This mimicked the different synaptic inputs these cells receive *in vivo*, with 10 Hz of glutamatergic input via the AMPA and NMDA receptor synapses, 8 Hz and 30 Hz (according to the synapse location) via the GABAergic input of fast GABA_A_ receptor synapses and 40 Hz via the slow GABA_B_ receptor synapses, see Supplementary Table 1. The time-course of the postsynaptic membrane conductances *g*_syn_(*t*) was modeled by a dual exponential function with time constants *τ*_1_ and *τ*_2_:

(1) $$g_{\text{syn}}\left(t\right)=g_{\max}\left(\exp\left(\frac{-t}{\tau_{1}}\right)-\exp\frac{\left(-t/\tau_{2}\right)}{\left(\tau_{1}-\tau_{2}\right)}\right)$$

[12, 27]. To model the synaptic noise, particularly that caused by the probabilistic nature of vesicle release [29, 30], synapses were switched to the stochastic regime under which *P*_*rel*⁡_ governs the probability that presynaptic events are translated into postsynaptic currents. This mode of synaptic operation allowed the *vesicle release noise* to be modeled in our simulations. To model the variation of vesicle quantal size and stochastic opening of postsynaptic ligand-gated channels, referred to as the *postsynaptic noise* throughout the paper, the synaptic conductances *g*_max⁡_ for each synaptic event were multiplied by a real number taken from normalized Gaussian distribution of a given coefficient of variation (*C*_*V*_), see Supplementary Table 1 for *C*_*V*_ of all synapses [31, 32].

### 2.2. Voltage-Gated Channel Noise

Six types of voltage-gated conductances are implemented in the model [46]. Cation channels Na, Ca, K_(DR)_, K_(AHP)_, K_(A)_, and K_(C)_ are localized on the somadendritic membrane. Na and K_(DR)_ channels localized on the axonal membrane employ faster kinetics. Except for the K_(C)_ all channels act stochastically in our model. This is implemented using the Markov process channel kinetics [57, 58]. In this “*microscopic mode*” of channels, each voltage-gated channel is found in different configurations of the Markov diagram according to the immediate combinations of gates. For example, the Na channel can be found in 8 (2^3^) configurations based on the binary states of its gates (*m_0_m_0_h_0_; m_1_m_0_h_0_; h_0_m_0_m_1_; m_1_m_1_h_0_; m_0_m_0_h_1_; m_1_m_0_h_1_; m_0_m_1_h_1_; m_1_m_1_h_1_*). The K_(DR)_ channel can be found in 4 configurations (*n_0_n_0_; n_0_n_1_; n_1_n_0_; n_1_n_1_*). The channel gates transit stochastically from one configuration into another during simulation. At every time step, **δ*t,* the average number of channels transiting from one configuration into another one is given by the binomial distribution *P*(*N*conf_*i*_, *pi*, *j*), where *N*conf_*i*_ is the number of channels in the *i*th configuration. *p_i,j_* represents the probability that a channel will transit from the *i*th configuration to the *j*th configuration. For example, for the Na channel, the *i*, *j* = {1,…, 8}. *p_i,j_* can be expressed as a product of the timestep **δ*t* and the channel-specific, voltage-dependent forward and backward transition rates *α* and *β* (see Supplementary Table 1, section voltage-gated conductances). Only if all state variables (gates) in one channel attain the value of 1, the channel opens. Markov configurations of other channel types simulated in microscopic mode are derived from the gate configurations listed in Supplementary Table 1, section voltage-gated conductances, as well as in [46].

The following equation determines the current *I*_HH_ originating in voltage-gated channels in somadendritic membrane compartments:

(2) $$\begin{array}{cc} I_{\text{HH}} & =N_{\text{Na}\_\text{open}}\cdot g_{\text{Na}}\cdot \left(V-V_{\text{Na}}\right)\\ & \;+N_{\text{Ca}\_\text{open}}\cdot g_{\text{Ca}}\cdot \left(V-V_{\text{Ca}}\right)\\ & \;+N_{\text{K}(\text{DR})\_\text{open}}\cdot g_{\text{K}(\text{DR})}\cdot \left(V-V_{\text{K}}\right)\\ & \;+N_{\text{K}(\text{A})\_\text{open}}\cdot g_{\text{K}(\text{A})}\cdot \left(V-V_{\text{K}}\right)\\ & \;+N_{\text{K}(\text{AHP})\_\text{open}}\cdot g_{\text{K}(\text{AHP})}\cdot \left(V-V_{\text{K}}\right)\\ & \;+g_{\text{K}(\text{C})}\cdot \min\left(1.0,\frac{\left[{\text{Ca}}^{2+}\right]}{250}\right)\cdot \left(V-V_{\text{K}}\right), \end{array}$$

where *N*_HH_open_ represents the number of open channels in question and *g*_chan_ the single channel conductance. The last voltage and [Ca^2+^]-gated conductance K_(C)_ were not simulated in the *“microscopic mode”* because according to (2), see also Supplementary Table 1, the transition rates were not depending on [Ca^2+^]. However, for proper channel noise implementation, the transition rates were required to depend on [Ca^2+^]. Because of the strong evidence of its considerable modulatory role in information processing in the dendritic tree [59], the K_(C)_ was maintained but modeled in the *“macroscopic mode”* obeying the classical Huxley-Hodgkin formalism [60]. To model the faster dynamics of the AP generation in the axon, the axonal voltage-gated conductances were updated with a tenfold smaller **δ*t* and implemented with different gate configurations and faster kinetics ([46], see Supplementary Table 1):

(3) $$\begin{array}{cc} I_{\text{HH}} & =N_{\text{Na}\_\text{open}}\cdot g_{\text{Na}}\cdot \left(V-V_{\text{Na}}\right)\\ & \;+N_{\text{K}(\text{DR})\_\text{open}}\cdot g_{\text{K}(\text{DR})}\cdot \left(V-V_{\text{K}}\right). \end{array}$$

Transition rates, single channel conductances *g*_chan_, the [Ca^2+^] dynamics of the K_(AHP)_ and K_(C)_ currents and simulation steps **δ*t* are listed in Supplementary Table 1.

### 2.3. Simulation Setup

For each particular simulation setup, different types of neuronal noise were switched on and off, and the time precision of the output spike trains at the axonal initial segment was estimated. Output spike trains were elicited by given discharge patterns of presynaptic neurons of duration *T*_*s*_ seconds and repeated *N*_*Tr*⁡_ times to get enough trains (these are called sweeps or trials) for analysis (for the detailed parameters of the presynaptic spike generators see Supplementary Table 1). Each given presynaptic discharge pattern (governed by Poisson processes and generated before the simulations) was reloaded from memory before each sweep, and thus its randomness was separated from the Markov processes controlling the channel noise during sweeps. The Markov-process model was implemented by compiling custom C routines into the GENESIS simulator as follows: knowing the probability *p_i,j_*, and also the number of channels in given state *N*conf_*i*_.

1. For numbers of channels in simulated compartments smaller than 25, the number of transiting channels was calculated directly by cycling the Monte Carlo method *N*conf_*i*_ times.
2. For numbers of channels greater than 25, the direct Poisson method [61] was used in case *p_i,j_* ×  *N*conf_*i*_ that was less than 1.
3. In all other cases, the binomial distribution was approximated and the number of transiting channels drawn from a parametrized Gaussian distribution [57].

The random number generator used in our simulations was the Lagged Fibonacci Generator [61] that is a part of the standard Numerical Recipes Library compiled into GENESIS by default. The numerical integration method used was the exponential Euler method [62].

### 2.4. Estimation of Spike Time Precision

**Figure 1:**
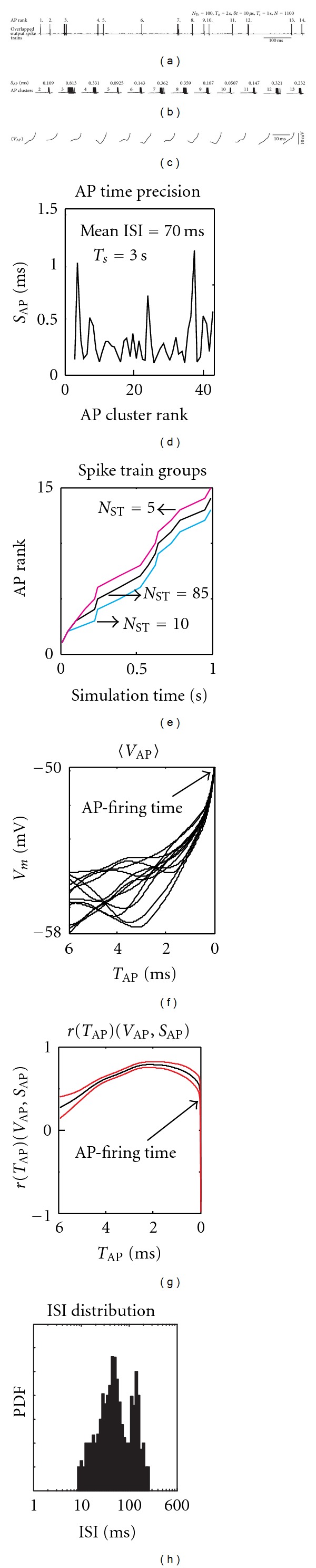
Analysis of AP clusters and estimation of AP time precision in the CA3 neuron. The effect of the channel noise. (a) Overlapped output spike trains generated by identical input presented to neuron *N*_*Tr*⁡_ times. (b) Extracted and overlapped shapes of APs-forming AP clusters. Values depicted on the top of each cluster show the *S*_AP_—the measure of AP time precision calculated as a standard deviation of AP times found in the AP cluster. (c) Shapes of 〈*V*_AP_〉*-*AP cluster's mean voltage course ending with triggering an AP. (d) *S*_AP_ of consecutive AP clusters for 3 s duration stimulation. (e) Visualization of spike train groups. Based on the Hamming distance between spike trains elicited by repeating the same input, the trains were sorted according to their firing pattern into different spike train groups (*N*_ST_ is the number of spike trains in each group). In this panel three groups are depicted by three lines differing in color. The ordinate shows the order of APs and the abscissa the firing time of AP. Despite the fact that each group contains different numbers of fired APs (some APs failed to be fired in individual trials), firing times in-between groups are well aligned. This indicates an interesting preference of the spiking mechanism to follow some pattern more frequently than others. (f) Aligned courses of 〈*V*_AP_〉 of different AP clusters. (g) Correlations *r*(*T*_AP_)(*V*_AP_, *S*_AP_) between voltages 〈*V*_AP_〉 of all AP clusters at given times *T*_AP_, and all clusters' *S*_AP_ were calculated under the channel noise effect. Correlation curves are delimited with their 0.95 confidence intervals (indicated in red), obtained from 40 correlation curves obtained over 40 various discharge patterns of presynaptic neurons repeated *N*_*Tr*⁡_ times. (h) The histogram demonstrates the common distribution of ISIs in our model.

The precision of spike timing was quantified using the measure *S*_AP_, calculated as the standard deviation of AP times collected by repeated identical stimulation. For very low levels of noise, where all output spike trains contained the same number of spikes, aligned synchronously in time, the *S*_AP_ was calculated as a standard deviation of AP times of the same AP rank (demonstration of such trains is depicted by panel (a) and (b) in Figure 1). This way of calculating the spike precision is referred to as the *AP rank method* throughout the paper. For low levels of noise, the AP clusters (accumulations of APs of aligned spike trains for a given discharge pattern of presynaptic neurons) were narrow and well separated in time. For higher noise levels spike trains in various trials differ too much from each other (containing isolated APs spread too far from expected AP clusters, or differing in numbers of fired APs, for example, in the case of AMPA or GABA_A_ postsynaptic noise). Therefore, for high noise levels, the *AP rank method* did not yield a relevant measurement of spike time precision.

Therefore, we chose another method, based on the Hamming distance. The purpose of calculating the Hamming distance was to collect suitable spike trains which were then processed by the *AP rank method*. We sorted similar spike trains into *spike train groups*, containing trains of mostly equal numbers of fired APs and with reduced numbers of extremely scattered spikes. The bin size used to quantize spike trains for Hamming distance calculations was 2 ms and the value of threshold Th_STG_ (calculated for a single AP), determining into how many different spike train groups the spike trains will be sorted, ranged from 0.1 for low levels of noise, to 0.3 for high levels of neuronal noise [63]. The bin size of 2 ms allowed us to group together spike trains having most of their APs scattered by less than 2 ms and to separate trains of different spiking patterns (see Figure 1(e)); according to our analysis, the spiking patterns separated by a Hamming distance less than Th_STG_ had mostly the same number of APs and matched highly conservative firing pattern. This was opposed by spiking patterns separated by a distance greater than threshold Th_STG_, differing in numbers of fired APs and also firing patterns (see Figure 1(e) for demonstration).

Once we separated spike trains into different groups, the *S*_AP_ value within a particular group increased monotonously with the level of neuronal noise. The resulting AP time precision was calculated by averaging the AP clusters' standard deviations *S*_AP_ over all *spike train groups*.

For even higher noise levels (e.g., simulating vesicle release noise, or postsynaptic noise of AMPA and GABA_A_ synapses) spike trains broke up into a lot of different spike train groups which did not contain enough trains for proper spike time analysis. However, spikes did not occur completely randomly but were still prone to occur in clusters (see Figure 3(a)). Thus, to analyze them, all spike trains for a given input pattern were superimposed, and AP clusters were found using a 10 ms wide time window, sliding along overlapped trains and collecting only those AP clusters containing at least 70% APs out of the *N*_*Tr*⁡_ value (see inset in Figure 3(a) for illustration of the method). The 10 ms window was chosen because in our estimates the measured separation between AP clusters exceeded 10 ms in more than 95% of cases, analyzing thereby APs found within a single AP cluster mainly. The 70% limit chosen was set in order to collect relevant amount of APs needed for further calculation. In this way we avoided processing of APs taken from less probable and mutually more separated *spike train groups.* The influence of extreme responses in spiking behavior was reduced this way as well. Finding the AP clusters this way and calculating respective values of *S*_AP_ is referred to as the *cluster method* throughout the paper.

#### 2.4.1. Setting the Model to the Stationary Conditions

Before the neuron was driven repeatedly with identical stimuli *N*_*Tr*⁡_ times, it was stimulated once by the adaptation input sequence lasting 2 s. This was done to reach steady-state values for the model's variables and in particular the intracellular calcium concentrations in respective compartments. The complete state of the model was recorded, once these initial steps were carried out.

#### 2.4.2. Synaptic Input, Input Synchrony

For various simulation arrangements the total number of presynaptic neurons varied (*N* = 110, 1100, 11000), whereas the total number of attached synapses remained fixed. This allowed us to vary the number of synapses driven by one presynaptic neuron (see Supplementary Table 1) and test the effect of synchrony of presynaptic neurons. Throughout various simulations the effect of *P*_*rel*⁡_, *C*_*V*_, the contribution of voltage-gated channel noise, and the effect of the stimulation pattern on AP time precision was estimated.

## 3. Results

### 3.1. Voltage-Gated Channel Noise

First we investigated the effect of voltage-gated channel noise on AP time precision. Initially the steady-state of the model was achieved by stimulating it with an adaptation input sequence lasting 2 s, after which the complete state of the model was saved. As the next step, the model was stimulated repeatedly 30 times (*N*_*Tr*⁡_ = 30; *T*_*s*_ = 1 s, or 3 s). The simulation always started from the steady state-values of the model variables retrieved from the adaptation sequence. The output spike trains were recorded and sorted into different spike train groups (see Figure 1(e) for an example of spike time group). Then the AP time precision was estimated by averaging the AP clusters' standard deviations *S*_AP_ over all *spike train groups*, as explained in Methods.

#### 3.1.1. Sub-Threshold Voltage Fluctuations

**Figure 2:**
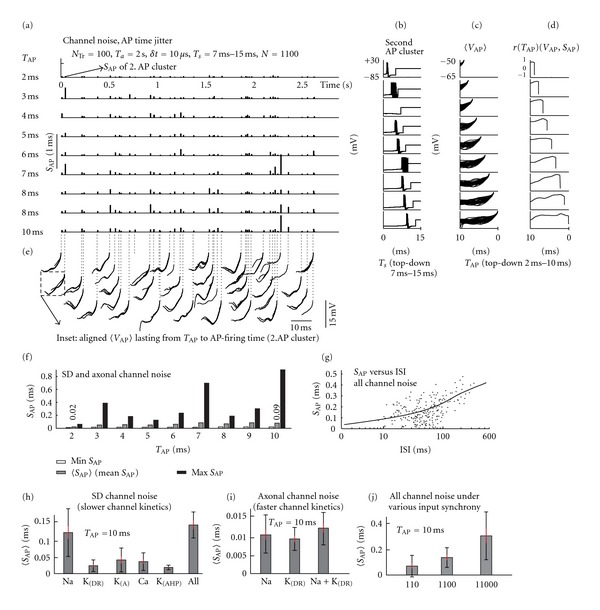
Voltage-gated channel noise and the effect of system memory of the model. (a) States of the model and its input were resumed as they were at times *T*_AP_ = {2,3, 4,5, 6,7, 8,9, 10} before the expected occurrence of each AP cluster and simulated for periods lasting *T*_*s*_ = {7,8, 9,10,11,12,13,14,15} ms repeatedly *N*_*Tr*⁡_ times (*N*_*Tr*⁡_ = 100) for each AP cluster. Bar heights in each of nine traces represent AP clusters' *S*_AP_ values for increasing value of *T*_AP_ (top-down). (b) An example of superimposed voltage AP courses of the second AP for increasing values of *T*_*s*_ (top-down). The traces end up at the end of simulation segments lasting *T*_*s*_. (c) Aligned 〈*V*_AP_〉 courses taken from all AP clusters merged altogether. (d) Correlations *r*(*T*_AP_)(*V*_AP_, *S*_AP_) between *S*_AP_ values and 〈*V*_AP_〉 courses of different intervals *T*_*s*_. (e) *V*_AP_ courses obtained under varying *T*_AP_ are aligned here for each AP cluster. The small misalignment of 〈*V*_AP_〉 shapes of given AP cluster is due to the voltage-gated channel noise incurring a small scattering of the resulting AP times collected under various durations of *T*_*s*_ simulation segments. (f) Relationship between the *T*_AP_ and estimated minimal, mean and maximal *S*_AP_ value with both somadendritic (SD) and axonal channel noise switched on. (g) The LOWESS quadratic fit matching the dependence between *S*_AP_ values and interspike interval ISI, constructed by processing the data from 30 s long continuous stimulations. The fitted curve shows the average value of *S*_AP_ for each given ISI (the effect of variable trajectory 〈*V*_AP_〉 influencing the AP time precision is averaged by the fitting procedure). (h) The effects of various types of somadendritic voltage-gated channel noise on AP time precision. (i) The effect of axonal initial segment voltage-gated channel noise on AP time precision. (j) Mean value of *S*_AP_ for various numbers *N* (on abscissa) of engaged presynaptic neurons mimicking various levels of synchrony among presynaptic neurons is demonstrated here. In panels ((h), (i), and (j)), the 95% confidence interval is indicated by red bars (obtained from simulations generating from 30 to 40 AP clusters). At the top of each bar, the black bars represent the ± standard deviation of *S*_AP_ values obtained over all AP clusters.

**Figure 3:**
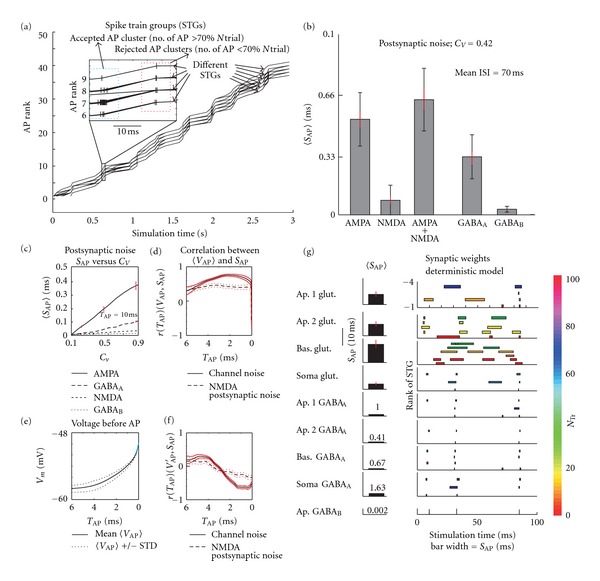
The effect of synaptic noise of various synaptic types. (a) Visualization of spike train groups analyzed under synaptic noise. The ordinate represents the AP rank of APs fired at times shown on abscissa. The braided lines in the main graph represent different spike trains of individual trials and demonstrate an interesting preference of the spiking mechanism preserving AP times well aligned despite different numbers of fired APs in trials. The inset focuses on the region of an AP cluster, demonstrating the cluster method used to analyze APs: the dotted blue rectangle in the inset represents the 10 ms time window moving along aligned spike trains and collecting only those AP clusters having within the 10 ms window number of spikes larger than 70% the value of *N*_*Tr*⁡_. (b) Resulting 〈*S*_AP_〉 estimated using 3 s stimulation duration with different types of synapses (mean ISI = 70 ms). For NMDA channel the *C*_*V*_ = 0.65, for other channels the *C*_*V*_ = 0.42. At the top of the bar the ± STD of *S*_AP_ is shown by black bar size. (c) 〈*S*_AP_〉 for different synaptic *C*_*V*_ and synapse type in question was calculated for values of *T*_AP_ = 10, locking the ISI at 10 ms. Higher values of ISI would yield larger *S*_AP_ as is the case in panel (b) in which the mean ISI = 70 ms. (d) Correlations curves *r*(*T*_AP_)(*V*_AP_, *S*_AP_) for neurons exhibiting channel noise only and postsynaptic NMDA noise only. Each correlation curve is delimited by 0.95 confidence interval. (e) The mean 〈*V*_AP_〉 curve obtained by averaging 〈*V*_AP_〉 over all AP clusters, delimited by the 〈*V*_AP_〉 ± standard deviation curves STD illustrating the range of 〈*V*_AP_〉 fluctuations over AP clusters. (f) Correlation curves *r*(*T*_AP_)(*V*_AP_, *S*_AP_) calculated by taking the time derivative of 〈*V*_AP_〉 are drawn here for channel noise only and postsynaptic NMDA noise only separately. Each correlation curve is delimited by 0.95 confidence interval. (g) Effect of synaptic weights. The effect of different synaptic types and their location on spiking variability in deterministic model (simulated without any membrane noise) is demonstrated by top-down graphs to the right, containing horizontal bars of various widths and colors. The color is mapped by the color bar to show the number of spike trains *N*_ST_ held in each spike train group, the widths in milliseconds represent *S*_AP_ of AP clusters in the group. (Compared to the channel noise effect analysis, the larger threshold Th_STG_ = 0.3 separating trains into different group was used here. The number *N* of all independently acting presynaptic neurons was 1100 with 110 neurons driving each individual synaptic subgroup—see Supplementary Table 1). In panels (b), (c), (d), (f), and (g) the red lines or vertical bars represent the 95% confidence interval.

The *S*_AP_ values for 3 s duration stimulations are shown in Figure 1(d). The *S*_AP_ of different AP clusters vary, depending mainly on the voltage course 〈*V*_AP_〉, recorded at the axonal initial segment, preceding the firing of the AP. The 〈*V*_AP_〉 is obtained by averaging over all cluster's APs voltage segments starting 6 ms before firing and ending up at the AP triggering point, set at the threshold voltage of −50 mV.  Figure 1(c) illustrates the 〈*V*_AP_〉 of respective AP clusters and Figure 1(f) their alignment (see also Figure 3(e) showing the mean 〈*V*_AP_〉 curve obtained by averaging 〈*V*_AP_〉 over all AP clusters). To analyze the role of the 〈*V*_AP_〉 trajectory on the AP precision quantitatively, the Pearson correlation *r* between AP clusters' voltages 〈*V*_AP_〉 at given times *T*_AP_ and the AP clusters' *S*_AP_ was calculated. Briefly,

(4) $$r\left(T_{\text{AP}}\right)\left(V_{\text{AP}},S_{\text{AP}}\right)=\langle \langle V_{\text{AP}}\rangle \left(T_{\text{AP}}\right)i,S_{\text{AP}}i\rangle ,$$

where 〈*V*_AP_〉(*T*_AP_)*i* is a vector containing AP clusters' 〈*V*_AP_〉 voltages at given times *T*_AP_, *S*_AP_ *i* is a vector containing AP clusters' *S*_AP_ values,  *i* = {1, …, *N*_AP_}, where *N*_AP_ is the number of fired AP clusters, and the double angular bracket denotes the Pearson correlation function. This correlation *r*(*T*_AP_)(*V*_AP_, *S*_AP_), calculated for channel noise only, is drawn in Figure 1(g). The time-course of the correlation reveals at what time *T*_AP_ before AP-firing the voltage of the axonal initial segment influences the AP time precision most. We found a small enhancement around 2 ms with quite a wide distribution before the AP firing time (see Figures 1(g), 2(d), and also Figure 3(d) comparing channel noise with NMDA noise). This curve indicates that voltages around 2 ms before firing an AP could be more influential than other voltages, at least in so far as it concerns the firing precision of our model. We also calculated the Pearson correlation *r* between the values of the time derivative of 〈*V*_AP_〉 and *S*_AP_, to find how the steepness of membrane voltage change influences *S*_AP_ (Figure 3(f), channel noise). We found a positive correlation until 3 ms before AP-firing time, and a negative correlation until the triggering of the AP. It seems that these rapid voltage changes occurring up to about 3 ms before the AP increase the *S*_AP_ (decrease the AP precision), whereas subsequent rapid voltage changes decrease the *S*_AP_ (improve the AP precision). Calculating the *r* with the absolute value of the 〈*V*_AP_〉 derivative produced nearly the same shape (not shown).

#### 3.1.2. System Memory of the Model

In the above-mentioned simulations, the neuron was driven by continuous firing of presynaptic neurons lasting up to a few seconds. Despite the identical stimulation used in trials, the model states and its firing differed among trials due to the varying seed used for channel noise. Thus, not only the immediate stimulation pattern eliciting the APs formed the actual firing pattern but also the history of firing along a particular trial course. To find out how this feature of system memory influences spike precision, we prepared a special simulation arrangement by means of which we were able to vary the duration of the stimulation period preceding the firing of individual APs.

Complete states of the model were recorded and saved as they were at times *T*_AP_ = {2,3, 4,5, 6,7, 8,9, 10} ms ahead of each *AP cluster time*. Then, for each AP cluster separately, the simulation was resumed (with the pattern of neuronal input resumed as well) *T*_AP_ ms ahead of the AP cluster and simulated *N*_*Tr*⁡_ times with simulations lasting *T*_*s*_ = {7,8, 9,10,11,12,13,14,15} ms. The simulation arrangement is described in detail in Figure 2 by panels (a), (b), and (c). The bar heights in each of nine traces in panel (a) represent the values of *S*_AP_ of AP clusters obtained by simulation sweeps lasting from 7 ms to 15 ms. Panel (f) summarizes the resulting *S*_AP_ by demonstrating the relationship between the period *T*_AP_ and *S*_AP_.

We found that the 〈*S*_AP_〉 (values of *S*_AP_ averaged over all AP clusters) increases with the *T*_AP_ and approaches 0.1 ms for *T*_AP_ = 10 ms (see Figure 2(f)). This is in agreement with *S*_AP_ values taken from the fitted curve in Figure 2(g) for ISIs around 10 ms.

#### 3.1.3. Influence of Interspike Interval (ISI)

The curve in Figure 2(g) fits the dependence between the *S*_AP_ values and the ISIs obtained from continuous, unresumed simulations (*T*_*s*_ = 30 s, *N*_*Tr*⁡_ = 30). The curve was constructed using the LOWESS fitting method (locally weighted scatter plot smoothing [64]), which looks for local quadratic polynomials matching the resulting fit. The effect of variable 〈*V*_AP_〉 trajectory on the AP time precision was averaged out by the quadratic fit procedure, so the fitted curve represents the average *S*_AP_ values at a given ISI. Although the *S*_AP_ values in panel (f) and panel (g) in Figure 2 were obtained with different simulation procedures, it seems that the fitted curve in panel (g) can be considered as an extension of the curve in panel (f) for values of *T*_AP_ exceeding 10 ms, or conversely the curve in panel (f) as an extrapolation for ISIs lower than 10 ms (ISIs smaller than 10 ms were rarely observed in continuous simulations). This indicates that the time separating two adjacent spikes determines the precision of the second spike time equally as does the duration *T*_AP_ of the interval ahead of the spike at which the simulation was resumed. Thus, with the at times *T*_AP_-resumed simulations, we were able to lock the ISI at a desired value while analyzing other effects unaffected by ISI.

#### 3.1.4. Contribution of Particular Channel Types

To separate the effect of different types of *voltage-gated channel noise* on AP time precision, we performed simulations where only a selected type of channel was noisy (*microscopic mode*), whereas others were noiseless (*macroscopic mode,* seeSection 2). We took advantage of the above-mentioned simulation arrangement used to analyze the model system memory and rerun the simulations 2 ms or 10 ms before expected AP times. In Figure 2 the effect of different types of voltage-gated channel noise is shown in panel (h) for the somadendritic membrane channel noise (axonal noise was switched off) and in panel (i) for the axonal membrane channel noise. Comparing the values of *S*_AP_, the influence of the somadendritic channel noise was larger than that of the axonal initial segments by nearly an order of magnitude.

#### 3.1.5. Input Synchrony

**Figure 4:**
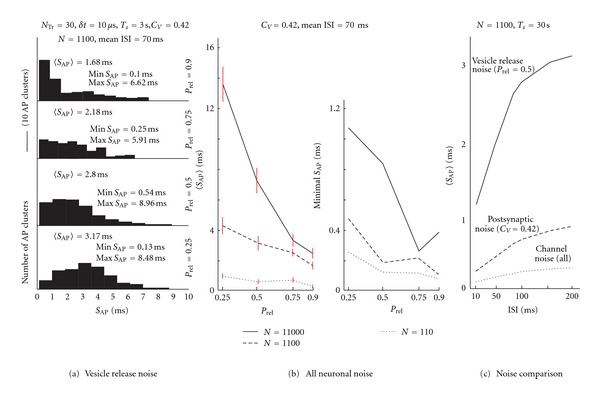
Vesicle release noise effect and the effect of all types of noise on the AP time precision. (a) *Vesicle release noise*. Four histograms represent the distribution of AP clusters' *S*_AP_ under various values of *P*_*rel*⁡_. Longer simulations yield more extreme values of max and min *S*_AP_-prolonging the simulation 10x (from 3 s to 30 s) the max *S*_AP_ increased by about 70%, and the min *S*_AP_ was approximately halved. Increasing the *T*_*s*_ once again to 300 s the max *S*_AP_ increased by only 20% whereas the value of min *S*_AP_ decreased by less than 10%. (b) *All neuronal noise.* The AP time precision of CA3 model with all noise sources switched on and relevant parameters set up to match real neurons. The effect of *P*_*rel*⁡_ and numbers of engaged presynaptic neurons* N*, representing different amount of synchronicity between presynaptic neurons, is shown here. The mean precision value 〈*S*_AP_〉 is shown to the left, and the minimal value of *S*_AP_ found is shown to the right. (c) Final comparison between various noise sources drawn for range of interspike intervals ISI fired in our model. In panel (b) the red vertical bars represent the 95% confidence interval of 〈*S*_AP_〉.

We also investigated how the synchronicity between presynaptic neurons influences the AP time precision. As explained in Methods, the synchronicity varied when engaging different numbers of presynaptic neurons *N* connected through a fixed number of attached synapses. The 〈*S*_AP_〉 for increasing number *N* of engaged presynaptic neurons is shown in Figure 2(j) and demonstrates how the effect of channel noise can be augmented by decreasing the input synchrony. We also tested the effect of input synchrony in the model running all noise sources. The result is drawn in Figure 4(b). Comparing the values of *S*_AP_ under synchrony levels ranging through different values of *N* = 110, 1100, 11000, spanning two orders of magnitude, the AP precision decreases by about ten times. It is obvious that the effect of input synchrony on the AP precision can be observed only in the case when some noise is present in the model; otherwise zero, AP dispersion would be obtained by stimulating the model with identical input.

### 3.2. Synaptic Noise

First we analyzed the effect of *postsynaptic noise *caused by the variation in vesicle quantal size. The *vesicle release noise* imposed by the probabilistic nature of synaptic vesicle release (see Section 2) was analyzed afterwards. Both types of noise were selectively switched on and off in different synapse types and their groups.

#### 3.2.1. Postsynaptic Noise

 As opposed to the spike trains generated with channel noise, the spike trains elicited after inclusion of postsynaptic noise were more variable, which may be attributed to the higher levels of noise produced mainly by AMPA and GABA_A_ postsynaptic channels. Therefore a sufficiently large spike train group, containing enough spike trains for *S*_AP_ estimates, could not be gathered (see the corresponding spike train groups in Figure 3(a)). In order to obtain AP clusters large enough for a reasonable *S*_AP_ analysis, all spike trains were superimposed in time. This revealed a good time alignment of APs from different spike train groups. The analyzable AP clusters were then obtained by sliding through the aligned spike trains with a time window of width 10 ms and collecting only those AP clusters containing at least 70% APs out of the *N*_*Tr*⁡_ value (see Section 2 and Insets in Figure 3(a)).

The resulting 〈*S*_AP_〉 of all AP clusters is shown separately for individual types of synapses in Figure 3(b). The used coefficient of variation of vesicle size was *C*_*V*_ = 0.65 for the NMDA channel and *C*_*V*_ = 0.42 for other channels [65–67]. The *S*_AP_ of NMDA and AMPA synapses switched on together is also shown. The 〈*S*_AP_〉 for different *C*_*V*_ values of AMPA, GABA_A_, GABA_B_, and NMDA synapses is shown in Figure 3(c) for ISI locked at 10 ms (achieved by resuming simulations *T*_AP_ = 10 ms before APs). Taking into account equal numbers of implemented AMPA and NMDA synapses, their identical spatial distribution and also the same characteristics of their presynaptic input, an order of magnitude larger effect of AMPA postsynaptic noise could be attributed to its five times bigger postsynaptic conductance *g*_max⁡_ (see Supplementary Table 1 and [43, 47, 65]) and also its smaller time constants *τ*_1_, *τ*_2_ compared to the NMDA synapse. Similarly, the effect of GABA postsynaptic noise is determined by analogous parameters, along with the fact that their spatial distribution differs from that of implemented glutamatergic synapses.

#### 3.2.2. Subthreshold Voltage Fluctuations and Comparison with Voltage-Gated Channel Noise

Since we are aware of the voltage-dependent characteristics of the voltage-gated channel noise effect, we were looking for a noise type which has a similar effect on AP precision, but does not depend on membrane voltage. Analyzing the correlation *r*(*T*_AP_)(*V*_AP_, *S*_AP_) of such noise allows us to eliminate the voltage-dependent manner of channel noise inevitably altering the voltage course of the 〈*V*_AP_〉, and thus to estimate the effect of the 〈*V*_AP_〉 course void of voltage dependence. Therefore we compared the *r*(*T*_AP_)(*V*_AP_, *S*_AP_) calculated for NMDA postsynaptic noise with the *r*(*T*_AP_)(*V*_AP_, *S*_AP_) calculated for voltage-gated channel noise (Figure 3(d)). We found a small, but significant difference between these two curves. As opposed to the *r*(*T*_AP_)(*V*_AP_, *S*_AP_) of voltage-gated channel noise (modeled in *microscopic mode*), the *r*(*T*_AP_)(*V*_AP_, *S*_AP_) of NMDA postsynaptic noise is less voltage-dependent (voltage-gated currents modeled in *macroscopic mode*), and its peak is shifted to the left by around 2 ms. This could imply that without the voltage-gated noise, the AP precision is influenced mostly by voltage fluctuations around 3.5 ms ahead of spike firing. Conversely, for channel noise, voltages closer to the triggering time are more influential, around 2 ms ahead of AP, near the firing threshold, at which the actual number of open channels is the most decisive factor for the generation of an AP [35]. Going even closer to the AP-firing time, the effect of fluctuation of opened voltage-gated channels decreases, which can be attributed to the commencement of spike generation, diminishing the effect of noise on AP precision.

#### 3.2.3. Effect of Weights of Various Synaptic Types on Spike Time Variability in a Deterministic Model

It is reasonable to expect that the AP time jitter depends on the number of attached synapses belonging to each particular synaptic type. Therefore, we first estimated the influence of particular types of synapses and their locations on the spike variability in a deterministic model lacking any noise sources (see the scheme of the CA3 neuron for the distribution of attached synapses in Supplementary Table 1). We switched off synaptic noise (thus running the model without any noise sources) and varied only the discharge pattern of a selected group of presynaptic neurons by leaving the discharge pattern of other groups unchanged. The resulting spike time variability was calculated analogously to the precision of spike timing under the influence of noise, except for glutamatergic synapses for which the AP *cluster method *failed to collect enough analyzable AP clusters (the scattering of APs was much broader than the 10 ms time window). Therefore, only spike trains sorted into groups of *N*_ST_ > 5 were analyzed to obtain the AP clusters' *S*_AP_ values (for glutamatergic synapses less than 15% of the data—spike train groups of *N*_ST_ < 5 were thereby discarded; for other synapses less than 10% of the data was discarded; *N*_*Tr*⁡_ = 100). In Figure 3(g) the effect of different synaptic types and their location is shown by nine top-down graphs. The color of horizontal bars represents the number of spike trains *N*_ST_ in each group. The bar widths correspond to the AP clusters' *S*_AP_. It is important to note that the most significant effect on the output spike trains' variability was incurred by the glutamatergic synapses, whereas the effect of the GABAergic synapses was smaller by an order of magnitude. See black *S*_AP_ bars to the left averaging *S*_AP_ values over all spike train groups having *N*_ST_ > 5 and all AP clusters.

The main goal of analyzing the weights of synaptic types in deterministic model as described in the above-mentioned paragraph was to inspect and scale the effect of *postsynaptic* and *vesicle release noise* as depending not only on the noise power but also on the weights of used synapses, number of connections to the presynaptic neurons, and also on other parameters used to simulate respective synapses, including the values of *g*_max⁡_ and time constants *τ*_1_ and *τ*_2_.

#### 3.2.4. Vesicle Release Noise

After the effect of implemented synapse types and their location on the output spike trains variability was classified and scaled in deterministic model, the *vesicle release noise* was switched on in all synapse types, whereas the other types of noise were switched off. In Figure 4(a), the effect of the probability of synaptic vesicle release *P*_*rel*⁡_ on the AP time precision is illustrated by four histograms. The histograms represent the distribution of AP clusters' *S*_AP_ values during the 3 s duration stimulation. As *P*_*rel*⁡_ decreases, the histograms' means move towards higher *S*_AP_. At the same time *S*_AP_ maxima are preserved. Although an increase in *S*_AP_ under lower *P*_*rel*⁡_ is expected, the preservation of high *S*_AP_ values in the tails for more reliable vesicle release (*P*_*rel*⁡_ = 0.9) is interesting and indicates that even with a high probability of vesicle release, the model continues to fire low levels of highly dispersed APs.

The individual contributions of vesicle release noise of a particular synapse type can be scaled from the relative contributions of each synapse type's postsynaptic noise effect (see Figure 3(b)), and from the relative weight of each synaptic group, as demonstrated by the 〈*S*_AP_〉 bars in Figure 3(g).

### 3.3. All Noise Sources

Finally, all implemented noise sources were switched on, including the *voltage-gated channel noise*, the *postsynaptic noise *and the *vesicle release noise*. In Figure 4(b), the effect of *P*_*rel*⁡_ on the 〈*S*_AP_〉 and the minimal *S*_AP_ is shown for different levels of synchrony among presynaptic neurons. Parameters determining the most significant sources of noise are set according to the available data (*C*_*V*_ = 0.42, *P*_*rel*⁡_ = 0.5–0.75), and thousands of synapses attached to the CA3 pyramidal neuron receive their input from around a thousand independently acting presynaptic neurons (parameter *N*). Under these conditions, the time precision of CA3 neurons can be as small as 0.2 ms for steep subthreshold depolarizations. Slowly evolving depolarizations, holding the membrane near the threshold for a longer time resulted in a value of *S*_AP_ larger than 10 ms.

The Figure 4(c) summarizes the separate effects of simulated noise sources, estimated for a range of ISIs fired in our model and with other parameters set so as to match values corresponding to real CA3 neurons.

## 4. Discussion

In this study a detailed multicompartmental model of the CA3 hippocampal pyramidal cell was simulated, and the effect of various noise sources on the spike-time precision was explored. It is well known that neurons are able to generate APs with millisecond and submillisecond precision, which has been demonstrated by electrophysiological and other experiments in both subcortical [15–22] and cortical [68–70] regions. There was a debate about the spike-timing precision. Shadlen and Newsome [71] were on one side, arguing for imprecise spike times, Koch, [43], Buzsaki [72] and others were on the other side of the debate, defending precise spike times. The essence of this debate was the question, whether the precise spike timing in cerebral cortex conveys some meaningful information, or alternatively, only mean firing rate matters. Most of the arguments of the supporters of the imprecise spikes are quite sound. Yet their arguments stem from limitations of electrophysiological experimental techniques, which rely on time averaging of recorded signals. With the advance of recording techniques, new evidence has accumulated [72, 73] and demonstrates that the fine temporal structure of spike trains also carries meaningful information. Essential points of this debate have been summarized in [43]. Despite this newly accumulated evidence supporting a role of spike precision, the observed discharge patterns of cortical neurons can be highly irregular in both spontaneous and stimulus-evoked conditions. However, it is not completely clear to what extent that variability reflects faithful encoding of temporally varying synaptic input or noise inherent in the spike-encoding mechanism. The timing and reliability of neuronal output spike trains are functions of many input variables, including the PSC kinetics, the spatial distribution of synaptic contacts, and the discharge pattern of connected presynaptic neurons. This makes the processes involved in spike train generation difficult to study *in vivo* without the input variables being affected by available experimental techniques. Therefore, we took advantage of *in silico *computer models that allowed complete control of the experimental environment and iterated the CA3 neuron model such that its responses matched the spiking regimes of real hippocampal neurons, discussed in detail in [45, 46] from which our model was adopted and inspired. The model fired with frequencies ranging from 5 to 40 Hz and with a coefficient of variation of interspike intervals ISI *C*_*V*(ISI)_ = 0.85. (Most of neocortical cells exhibit a *C*_*V*_ greater than or equal to 1 [8].) The firing of the CA3 model in our simulations was also highly variable, which corresponds to the highly variable firing regimes found in real CA3 neurons and also with high firing variability of individual CA3 neurons during a single trial in behavioral tasks [25]. The discharge rates of our CA3 neuron ranged from theta rhythm band, 5 Hz [25, 74] and reached rates of about 40 Hz, approaching the gamma rhythm (30 to 80 Hz). Although hippocampal cells' discharge frequency is closer to the theta regimes and even lower [25], the frequencies around the gamma band were also observed in CA3 neurons [75] and were simulated in our model as well, allowing us to measure the spiking precision for shorter ISI intervals. To compare firing statistics with real data, the ISI histogram was constructed by collecting ISIs from longer stimulations (*T*_*s*_ > 3 s). Its shape resembles the biphasic course found in real CA3 pyramidal cells [25], see Figure 1(h)). The bursting regime was also reproduced by our model; however its spiking precision was not estimated, mainly due to difficulties when using any of the above-mentioned methods of spike train analysis (see Section 2). As was described in Methods, the core of the CA3 model was adapted from [45, 46] and further refined according to the available literature (see Supplementary Table 1). It is known that the CA3 pyramidal neurons are driven by different types of synaptic conductances being discharged at different frequencies. Therefore, the attached GABA_A_ and GABA_B_ inhibitory synapses were driven by frequencies mimicking either theta or gamma rhythms observed in these circuits [76]. The excitatory input delivered to the AMPA and NMDA receptor synapses discharged at 10 Hz in the model, and results of the simulation agree with the experimental findings [28]. Altogether close to 8000 excitatory and inhibitory synapses were simulated, and thus we believe that realistic numbers of synapses were modeled. Most of the implemented synaptic parameters, including vesicle release statistics, numbers of synapses, conductances, and PSC kinetics (see Supplementary Table 1 and References) were obtained from available literature describing CA3 cells and similar neurons.

We can conclude from our simulations that the AP time precision was mostly influenced by the synaptic noise incurred by the probabilistic nature of synaptic vesicle release (*vesicle release noise*) and to a lesser degree by the variation of vesicle quantal size (*postsynaptic noise*). The *voltage-gated channel noise *had the smallest effect on AP time precision (see Figure 4(c)).

In analyzing the individual contributions of particular types of voltage-gated channels, we found that the most influential ones were the Na channel (〈*S*_AP_〉 = 0.13 ms), the K_(A)_ channel (〈*S*_AP_〉 = 0.04 ms), and the K_(DR)_ channel (〈*S*_AP_〉 = 0.05 ms) of the somadendritic membrane. Axonal Na and K_(DR)_ voltage-gated channel noise effects were about one order of magnitude (〈*S*_AP_〉 = 0.01 ms) smaller. A similar study investigating the effect of channel noise indicates that the effect of channel noise on spike-timing precision could be about ten times greater (1.5 ms for all channels) than in our model (0.15 ms for all channels) [36].

For the *postsynaptic noise* the 〈*S*_AP_〉 was 0.6 ms for vesicle quantal size with a *C*_*V*_ = 0.42 [65, 67]. The overall effect of *postsynaptic noise* is, however, about four times lower than the effect of *vesicle release noise* incurring *S*_AP_ values varying from 1 ms to 7 ms, with a mean value of 2.5 ms (for probabilities *P*_*rel*⁡_ ranging from 0.5 to 0.75, these values were found in real synapses [29, 30]).

If all noise sources were switched on, the resulting average precision was about 3 ms (see panel (a) and panel (b) in Figure 4) and varied mostly based on values of *P*_*rel*⁡_ and *N* (the value determining the synchronicity among presynaptic events). Similar AP precisions (less than 2.45 ms) were assessed in electrophysiological experiments by injecting the current made up of synchronous excitatory PSC of fast kinetics [27]. Currents made up of slower excitatory PSC kinetics incurred the AP jitter higher than 6.58 ms [27]. For simulations in which all noise sources were switched on, a maximal AP precision of about 0.2 ms and the minimal precision of about 10 ms were reached, varying mostly upon the actual synaptic input shaping the subthreshold voltage course (measured for *P*_*rel*⁡_ = 0.75 and 1100 independent presynaptic neurons). The mean 2-3 ms precision 〈*S*_AP_〉 reached in our model matches the spike jitter of pyramidal cell axon collaterals as modeled in [38].

By constructing histograms depicting the distribution of *S*_AP_ values, it was found that by increasing the *P*_*rel*⁡_ values the model surprisingly continued to fire highly dispersed APs (demonstrated by preservation of histograms' maxima), whereas the mean histogram *S*_AP_ value decreased, as expected. This dispersion is attributed, as mentioned above, to the varying voltage course 〈*V*_AP_〉 of axonal initial segment preceding the firing of individual APs. It is well known that rapid depolarizing stimuli can elicit APs of much higher precision than slower ones [34, 68, 77] emphasizing the role of stimulus properties on the temporal precision of fired spikes [27]. This is commonly ascribed to neuronal noise, the effect of which increases proportionally with the time during which the membrane voltage is held near the threshold. This makes the spike generation mechanism more susceptible to noise at gradual depolarizations, under which even weak voltage perturbations can easily elicit or cancel APs. We tried to describe this effect quantitatively and calculated the *r*(*T*_AP_)(*V*_AP_, *S*_AP_), a measure relating the spike time precision with the time course of 〈*V*_AP_〉. The *r*(*T*_AP_)(*V*_AP_, *S*_AP_) shows how the trajectory of 〈*V*_AP_〉 determines the firing precision. We found that voltages around 4 ms–2 ms ahead of AP firing were most decisive for firing precision. Moreover, to find how the steepness of membrane voltage influences the *S*_AP_, we calculated *r*(*T*_AP_)(*V*_AP_, *S*_AP_) a correlation between the time derivative of 〈*V*_AP_〉, and *S*_AP_. We found that rapid voltage changes occurring up to about 3 ms before AP increase the *S*_AP_ (decrease AP precision), whereas subsequent voltage changes decrease the *S*_AP_ (improve AP precision).

In addition to the effect of the 〈*V*_AP_〉 trajectory, the interspike intervals ISI, separating adjacent APs, influenced the spike time precision considerably. We found that the firing precision varied by a factor of 10 for ISIs differing by a factor of 50 (for ISI = 2 ms the 〈*S*_AP_〉 was 0.02 ms, for ISI = 100 ms the 〈*S*_AP_〉 was 0.2 ms, see Figure 2(g)). By comparing the effects of ISI and 〈*V*_AP_〉, the latter was by about one order of magnitude larger (minimal *S*_AP_ = 0.012, maximal *S*_AP_ = 0.9, evaluated for ISI = 10 ms and channel noise, see Figure 2(f)).

Finally, the most prominent factor influencing the resulting effect of each modeled neuronal noise type was the level of input synchrony, implemented by varying the numbers of presynaptic neurons connecting to a fixed number of attached synapses. Input synchrony enhanced the resulting AP dispersion by more than tenfold, as measured by increasing the number of independently acting presynaptic neurons from 110 to 11000. Such results are naturally expected, since synchronous or coincident synaptic events commonly result in more precise firing of APs [78, 79]. However, some studies indicate that the noise effect improves signal transmission fidelity in some population coding schemes by decreasing the synchronicity of firing neurons [80].

Alternative methods to study the effect of neuronal noise on AP timing and firing regimes have been used in modeling the cerebellar granule cell excitability [81], in modeling the channel noise in detailed model of hippocampal CA1 pyramidal neuron [36], or in estimating the effect of channel noise on precision of spike propagation along axons [38].

## 5. Conclusion

To summarize, spike coding precision was highly variable during stimulations and depended mostly on the actual spatiotemporal pattern of neuronal input determining the subthreshold voltage course, on the probability of synaptic release, on the degree of synchronicity of synaptic inputs and also on the value of the interspike interval. Regarding the noise sources (see Figure 4(c) for comparison).

1. The most influential was the noise incurred by the probabilistic nature of synaptic vesicle release (*vesicle release noise*) of spike time precision (*S*_AP_) varying from 1 ms to 7 ms, with a mean value of 2.5 ms.
2. Of the second importance was the noise incurred by vesicle quantal size variation (*postsynaptic noise*) causing the mean *S*_AP_ value to vary from 0.03 ms to 0.6 ms, depending on synapse type in question.
3. The least influential was the *voltage-gated channel noise*, causing the mean *S*_AP_ values to range from 0.02 ms to 0.15 ms.

When all noise sources were switched on together, the resulting average precision was about 3 ms (see panel (a) and panel (b) in Figure 4). The minimal and maximal precision depended mostly on the subthreshold voltage course, causing the *S*_AP_ value to be as high as 10 ms for very slow voltage changes, and decreasing it to even 0.2 ms for voltages of very high steepness. Moreover, we found that the *S*_AP_ was rising with rising ISI (see Figure 4(c)).

The precision and reliability of real CA3 hippocampal neurons in unconstrained and freely behaving animals have not yet been fully elucidated. Much less is known about the time precision of the majority of cortical cells participating in similar nonsensorial tasks. Even if the precision of CA3 pyramidal neurons correlates with our estimates, the question concerning the biological relevance of such variable timing precision would naturally arise. High variations of AP precision during stimulation lasting seconds, or during a single behavioral task, depreciate the effectiveness of temporal coding. It is reasonable to assume that neurons in the next computational layer expect APs occurring with declared precision and are prone to process information encoded by that timing. Since for individual neurons the value of the relevant precision of individual spikes is not accessible, the processing of trains of variable time precision can have two possible outcomes. Namely, the underestimation of delivered information in the case for which the neurons are adapted to low temporal resolution or the misinterpretation of information in the case for which they are adapted to high temporal resolution when decoding random AP timing. It seems thus that only spike trains of constant AP time precision could effectively be deciphered by individual neurons. However, in cases when the population code is used, the circuits (detecting the amount of AP coincidence between functionally similar neurons sharing the same input and generating individual spikes of similar time precision) could effectively process such variable time code by detecting the exact temporal separation between spikes originating in functionally identical neurons. Such neuronal circuits are well known in the auditory pathway of many animals and it is possible that similar hardware detecting the precise coincidence might also be found in nonsensorial structures such as those in the hippocampus [78].

## List of Abbreviations, Definitions, and Symbols

*a*, *b*, *c*, *h*, *m*, *n*, *q*, *s*: Dimensionless state variables, also called channel gate variables, taking either binary values of 0 or 1 for the “microscopic mode” simulations exhibiting the channel noise, or continuous values between 0 and 1 for the “macroscopic mode” simulations void of channel noise,
AMPA: 2-Amino-3-hydroxy-5-methyl-4-isoxazole-propionate acid type of synapse
ap., bas.: Abbreviations for apical or basal dendrites
AP(s): Action potential(s)
AP clusters: Accumulations of the APs in overlapped spike trains
*C*_*V*_: Coefficient of variation (standard deviation divided by mean) of *g*_max⁡_ currents of particular synapse
[Ca^2+^]: Concentration of Ca^2+^ ions in membrane compartments
CA3: The third area of the hippocampal Cornu Ammonis
*D*_syn_: Peak density of synapses
*δt*: Simulation step
*E*_syn_: Equilibrium potential of synapse
GABA: Gamma-amino-butyric acid type of synapse
*g*_max⁡_: Peak conductance of single postsynaptic conductance (PSC)
*g*_syn_: Actual synaptic conductance
HH: Hodgkin-Huxley
ISI: Inter spike interval
*I*_HH_: (The Hodgkin-Huxley) current in voltage-gated channels
*L*_syn_: Distance separating the localization of peak synaptic density and the localization of soma (in meters)
*N*: Number of presynaptic neurons
*N*conf_*i*_: Number of channels in the *i*th configuration
*N*_HH_open_: Number of open voltage-gated channels of particular HH type
*N*_ST_: Number of output spike trains in the identified spike train group
*N*_*Tr*⁡_: Numbers of repeated stimulation (sweeps) with identical input (discharge pattern of presynaptic neurons)
NMDA: N-methyl-D-aspartate type of synapse
*N*_AP_: Number of AP clusters in given simulation
〈*V*_AP_〉: The voltage course obtained by averaging over voltage courses preceding the firing of APs in particular AP cluster
*p*_*i*,*j*_: Probability that the channel will transit from the *i*th configuration to the *j*th configuration
*P*_*rel*⁡_: Probability of mediator release—formation of postsynaptic potential, subsequent to the arrival of presynaptic spike
PSC: Postsynaptic conductance
*r*(*T*_AP_)(*V*_AP_, *S*_AP_): The Pearson correlations between voltages 〈*V*_AP_〉 of all AP clusters at given times *T*_AP_ and all clusters' *S*_AP_ values, see (4)
*S*_AP_: Standard deviation of AP times found within the AP cluster
〈*S*_AP_〉: Values of *S*_AP_ averaged over all AP clusters
Syn_*σ*_: Standard deviation of synaptic density distribution along dendrites (in meters)
SD: Somadendritic (membrane) compartments
Sat*g*_max⁡_: Single synapse conductance saturation
STG: Spike train group, a group containing trains of similar spike patterns
*τ*_1_, *τ*_2_: Postsynaptic potential raise and decay time constants
Th_STG_: Threshold for classifying spike trains into different groups
*T*_AP_: Time before firing APs
*T*_*s*_: Duration of one simulation run (trial, sweep)
*V*: Membrane potential
*V*_Na_, *V*_Ca_, *V*_K_: Reversal potentials of respective ions (absolute values).
